# Intrinsic Bulk Quantum Oscillations in a Bulk Unconventional Insulator SmB_6_

**DOI:** 10.1016/j.isci.2020.101632

**Published:** 2020-10-01

**Authors:** Máté Hartstein, Hsu Liu, Yu-Te Hsu, Beng S. Tan, Monica Ciomaga Hatnean, Geetha Balakrishnan, Suchitra E. Sebastian

**Affiliations:** 1 Cavendish Laboratory, Cambridge University, Cambridge CB3 0HE, UK; 2 Department of Physics, University of Warwick, Coventry CV4 7AL, UK

**Keywords:** Condensed Matter Physics, Quantum Solids, Strongly Correlated Electron Systems

## Abstract

The finding of bulk quantum oscillations in the Kondo insulator SmB_6_ proved a considerable surprise. Subsequent measurements of bulk quantum oscillations in other correlated insulators including YbB_12_ lent support to our discovery of a class of bulk unconventional insulators that host bulk quantum oscillations. Here we perform a series of experiments to examine evidence for the intrinsic character of bulk quantum oscillations in floating zone-grown single crystals of SmB_6_ that have been the subject of our quantum oscillation studies. We present results of thermodynamic, transport, and composition analysis experiments on pristine floating zone-grown single crystals of SmB_6_ and compare quantum oscillations with metallic LaB_6_ and elemental aluminum. These results establish the intrinsic origin of quantum oscillations from the insulating bulk of floating zone-grown SmB_6_. The similarity of the Fermi surface in insulating SmB_6_ with the conduction-electron Fermi surface in metallic hexaborides is at the heart of a theoretical mystery.

## Highlights

• No metallic inclusion contribution to quantum oscillations in ultrapure insulating SmB_6_
• Unconventional low energy excitations responsible for bulk quantum oscillations in SmB_6_
• Insulating SmB_6_ Fermi surface resembles conduction-e^-^ Fermi surface of metallic LaB_6_

## Graphical Abstract

**Figure:**
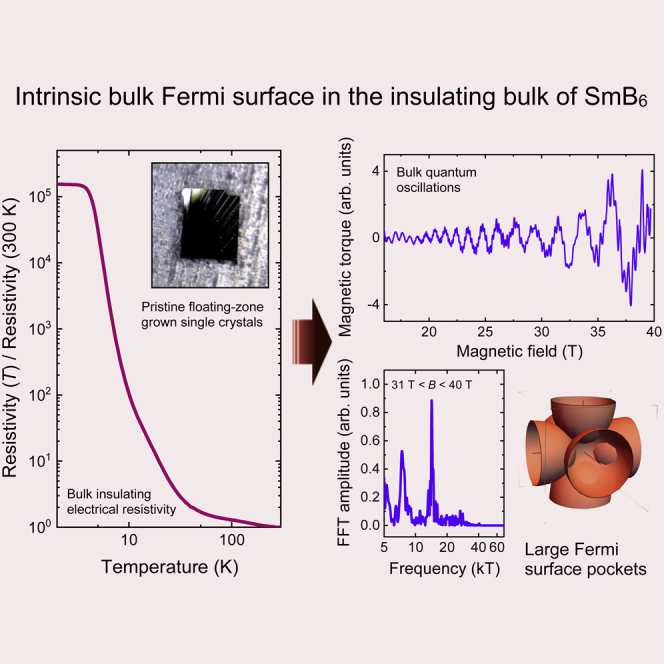

## Introduction

The *f*-electron bulk Kondo insulating system SmB_6_, first identified in Menth et al. (1969), and recently proposed to be a correlated topological insulator (Dzero et al., 2010), has been found to have a bulk insulating gap of size ≈ 3.5 meV through experimental characterization by numerous techniques, including infrared absorption, inelastic neutron scattering, optical conductivity, electron tunneling, intermediate-temperature specific heat capacity, and electrical resistivity (Antonov et al., 2004). Any consequences of crystal electric field effects on the band structure of this *f*-electron system are included in this experimentally determined bulk insulating gap (Baruselli and Vojta, 2016). The bulk insulating gap in single crystals of SmB_6_ is minimally affected by the applied magnetic field up to at least the highest accessible static magnetic field of 45 T (Tan et al., 2015; Wolgast et al., 2017).

Given the history of flux-grown samples of other systems (e.g. UBe_13_ (Wolf et al., 1991; Corcoran et al., 1993), CaB_6_ (Terashima et al., 2000)) in which inclusions have been shown to yield effects such as quantum oscillations, our original study (Tan et al., 2015) and our present study chose for measurement floating zone-grown single crystals of SmB_6_ which are not grown out of external flux. Our surprising finding of quantum oscillations in the magnetic torque of floating zone-grown single crystals of SmB_6_ (Tan et al., 2015), despite the bulk insulating gap, has given rise to multiple proposals for their potential origin. Physical mechanisms proposed as potential explanations for the observed quantum oscillations include bulk in-gap low energy excitations (Tan et al., 2015; Hartstein et al., 2018), two-dimensional surface conduction states (Li et al., 2014), tunneling across the bulk insulating gap (Knolle and Cooper, 2015, 2017), and impurity inclusions (Thomas et al., 2019; Fuhrman et al., 2018; Fuhrman and Nikolić, 2020), among others (Baskaran, 2015; Knolle and Cooper, 2015, 2017; Erten et al., 2016, 2017; Chowdhury et al., 2018; Sodemann et al., 2018; Thomson and Sachdev, 2016; Skinner, 2019; Peters et al., 2019; Liu and Balents, 2017; Shen and Fu, 2018; Valentine et al., 2016; Harrison, 2018; Zhang et al., 2016; Pal, 2017; Riseborough and Fisk, 2017; Grubinskas and Fritz, 2018; Sakhya and Maiti, 2020; Pixley et al., 2018; Ram and Kumar, 2017; Anderson, 1992; Coleman et al., 1993; Motrunich, 2006; Grover et al., 2010; Paul et al., 2007; Kishigi and Hasegawa, 2014).

In this paper, we present evidence for the intrinsic bulk origin of the observed quantum oscillations in floating zone-grown single crystals of SmB_6_ through a complement of measurements including optical emission spectroscopy, thermal transport, electrical transport, specific heat capacity, magnetic susceptibility, and a comparative study of quantum oscillations with elemental aluminum. We further establish the existence of bulk in-gap low energy excitations in the bulk unconventional insulator SmB_6_ by examining a range of experimental signatures in floating zone-grown single crystals, including the observation of high frequencies corresponding to a significant volume fraction of the Brillouin zone, large absolute amplitude of quantum oscillations of the order of magnitude expected from the entire bulk of the sample, growth of quantum oscillation amplitude at low temperatures, finite linear coefficient of specific heat capacity at low temperatures, and the similarity of the angular-dependent quantum oscillations observed in insulating SmB_6_ with those observed in the metallic hexaboride LaB_6_.

## Results

### Pristine Chemical Composition of Floating Zone-Grown Single Crystals of SmB_6_ Used for Quantum Oscillation Measurements

We demonstrate the high quality of floating zone-grown single crystals of SmB_6_ used for our quantum oscillation measurements and place constraints on any impurity content using a combination of experimental techniques. To quantify the bulk elemental composition of our samples, we perform a new, high accuracy, and precision elemental characterization measurement using inductively coupled plasma optical emission spectrometry (ICP-OES) (Jarvis et al., 1992). The bulk chemical composition analysis (shown in Table 1) indicates very high purity, with any extrinsic chemical content (including aluminum and gadolinium) other than tellurium and yttrium constrained below the level of detection (≈ 0.01%at in most cases).

**Table 1:** Inductively Coulped Plasma Optical Emission Spectroscopy Impurity Analysis

Parts per Million Range	Elements
Limit of detection (100)	Al, Au, As, Ba, Be, Bi, Ca, Cd, Ce, Co, Cr, Cu, Dy, Eu, Fe, Gd, Ge, Hf, Hg, Ho, Ir, K, La, Li, Lu, Mg, Mn, Mo, Na, Nb, Nd, Ni, Os, P, Pb, Pd, Pt, Rb, Re, Ru, S, Sb, Sc, Se, Si, Sn, Sr, Ta, Tb, Ti, Tl, Tm, U, V, Yb, Zn, Zr.
Limit of detection (400)	Ag, Er, Ga, Nd, Se.
100–1000	Te, Y.
1000–10,000	
10,000 or >1%at	B, Sm.

Limits on impurity concentration in parts per million in floating zone-grown SmB_6_ single crystals used for our quantum oscillation measurements (Hatnean et al., 2013) as found by inductively coupled plasma optical emission spectrometry (ICP-OES). A large quantity of sample used ≈50 mg enables higher detection accuracy and precision across a broad range of elements examined than had been previously reported. Extrinsic impurity content other than tellurium and yttrium is ruled out to within the detection limit (≈0.01%at in most cases).

**Figure 1:**
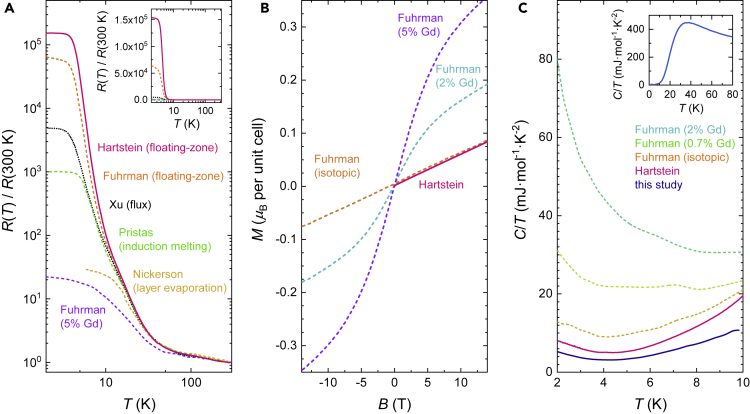
Comparison of Electrical Transport, Magnetic, and Thermal Properties of SmB_6_ Single Crystals (A) Electrical resistivity as a function of temperature on single crystals of SmB_6_ grown by different methods, normalized to the resistivity at 300 K. The inverse residual resistivity of new floating zone-grown single crystals of SmB_6_ used to measure quantum oscillations in this study reach a value of the order 10^5^, higher than the isotopic floating zone-grown sample with minimal rare-earth impurities from Fuhrman et al. (2018), and more than an order of magnitude higher than samples grown by other growth methods (flux (Xu et al., 2016), induction melting (Pristáš et al., 2014), layer evaporation (Nickerson et al., 1971)). The inset shows the same data plotted on a linear scale, further demonstrating the high inverse residual resistivity of our floating zone-grown single crystals. (B) Measured magnetization of Gd-doped SmB_6_ samples from a recent report by Fuhrman et al. (2018) (dashed lines in purple and cyan), showing a non-linear magnetization due to magnetic impurities. Measured magnetization of floating zone-grown single crystals of SmB_6_ used for our quantum oscillation studies shows linear paramagnetic behavior (Hartstein et al., 2018), as expected for SmB_6_ free from magnetic impurities (magenta line), also seen for an isotopic floating zone-grown sample from Fuhrman et al. (2018) (orange dashed line). We place an upper bound of ≈ 0.04%at on the magnetic impurity concentration of our sample by fitting with the Langevin function using an effective moment $\mu_{\text{eff}}=7.94$$\mu_{\text{B}}$ corresponding to the Gd^3+^ state (Fuhrman et al., 2018). (C) Measured specific heat capacity of floating zone-grown SmB_6_ single crystals used in our quantum oscillation measurements (blue and magenta lines (Hartstein et al., 2018; Hatnean et al., 2013)), floating zone-grown isotopic sample (orange dashed line (Fuhrman et al., 2018)) and flux-grown Gd-doped samples (green and cyan dashed lines (Fuhrman et al., 2018)). The finite linear coefficient of the specific heat capacity is seen to be ≈ 3mJ·mol^−1^·K^−2^ in the vicinity of 2 K for our highest purity floating zone-grown single crystals of SmB_6_ where the magnetic impurity concentration is limited to within ≈ 0.04%at, which is comparable to the finite linear coefficient of the specific heat capacity of isotopically enriched SmB_6_ also with similarly low magnetic impurity content (Gabáni et al., 2001, 2002b). The inset shows the measured specific heat capacity of a single crystal of SmB_6_ from the same growth as the main panel up to a high temperature of 80 K.

Suggestions of magnetic inclusions such as gadolinium in single crystals of SmB_6_ have been put forward based on studies of SmB_6_ single crystals impregnated with gadolinium impurities (Fuhrman et al., 2018; Geballe et al., 1970; Kim et al., 2014; Gabáni et al., 2002a, 2002b; Orendáč et al., 2017; Konovalova et al., 1982). Here we quantitatively evaluate the magnetic impurity content in our samples by comparing low temperature magnetization measurements of our single crystals of floating zone-grown SmB_6_ to the recently published impurity study by Fuhrman et al. (2018) to place limits on any magnetic impurity content. For a paramagnet such as SmB_6_, the magnetization is expected to be linear as a function of an applied magnetic field. Any magnetic impurities in a paramagnet would be expected to yield a deviation from linearity, described by the Langevin function. The extent of deviation from linearity may be used to place an upper limit on any magnetic impurity content, which has been used in the study by Fuhrman et al. (2018). Measurements on floating zone-grown single crystals of SmB_6_ used in our quantum oscillation measurements yield a linear magnetization (Hartstein et al., 2018), with a deviation from linearity consistent with a magnetic impurity concentration level limited to within 0.04%at (Figure 1B).

**Figure 2:**
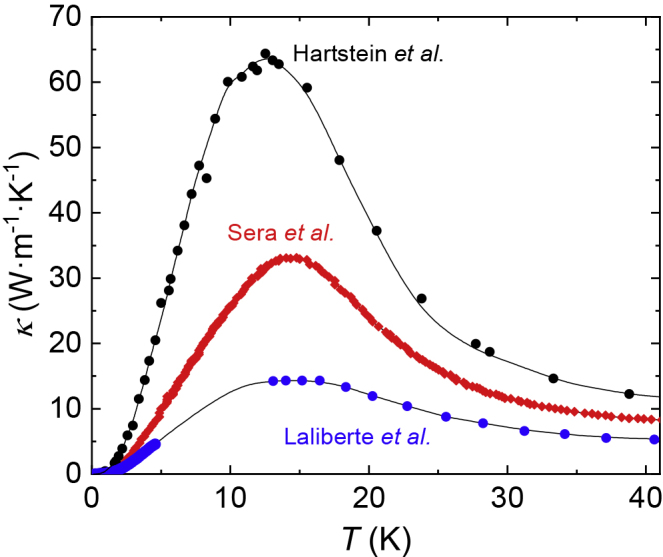
Comparison of High Temperature Thermal Conductivity Phonon Peak in SmB_6_ Single Crystals Thermal conductivity κ as a function of temperature for a floating zone-grown SmB_6_ single crystal from the same batch as single crystals on which we measure quantum oscillations (black circles (Hartstein et al., 2018; Hatnean et al., 2013)), compared to the thermal conductivity measured for floating zone-grown samples in previous studies (red diamonds (Sera et al., 1996), blue circles (Laliberte et al., 2018)). The size of the peak is found to be largest for the floating zone-grown crystal used in our study, indicating high sample quality.

The high-temperature phonon peak marks the regime where the thermal conductivity is limited by impurities and defects, and the size of this peak provides a measure of the mean free path, thus indicating sample quality. Figure 2 shows a larger high temperature phonon peak measured on our floating zone-grown SmB_6_ single crystals compared to those measured by other groups, indicating very high sample quality of our floating zone-grown single crystals (Hartstein et al., 2018). The considerably larger mean free path of our single crystals compared to samples from Sera et al. (1996); Laliberte et al. (2018); Boulanger et al. (2018) explains the boundary-limited phonon behavior observed in our single crystals of SmB_6_ which we use to conclude field-induced contribution to thermal conductivity (Hartstein et al., 2018). The difference in mean free path, inferred from high temperature thermal conductivity data, also potentially explains the non-boundary-limited phonon behavior reported in less pure single crystals of SmB_6_ (Laliberte et al., 2018). This comparison highlights the importance of sample purity in understanding the intrinsic behavior of SmB_6_.

**Figure 3:**
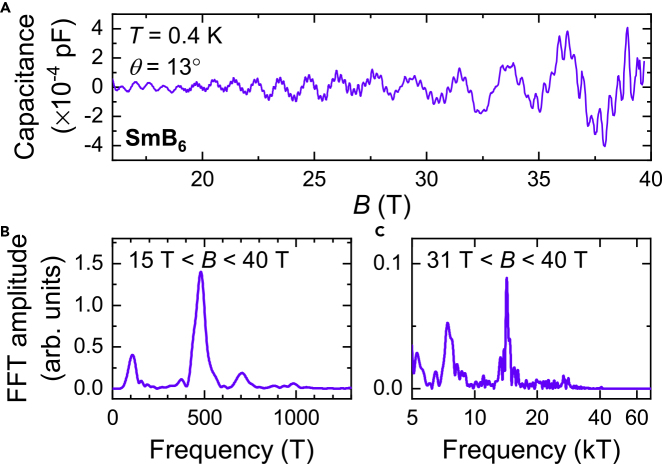
Magnetic Torque Quantum Oscillations in Pristine Floating-Zone Grown SmB_6_ Single Crystals (A) Quantum oscillations in the magnetic torque of floating zone-grown SmB_6_ single crystals measured in this study as a function of magnetic field. The field is aligned 16° from the [001] direction in the [001]-[111]-[110] rotational plane. (B and C) Fourier transforms of the measured magnetic torque as a function of inverse magnetic field in the field ranges (B) 15 T < *B* < 40 T and (C) 31 T < *B* < 40 T, revealing a wide range of quantum oscillation frequencies ranging from 300 T to 15 kT, similar to our previous study in Tan et al. (2015). A higher magnetic field window is used to reveal the high frequency oscillations corresponding to the α, λ and ξ branches identified in Figure 7A.

**Figure 4:**
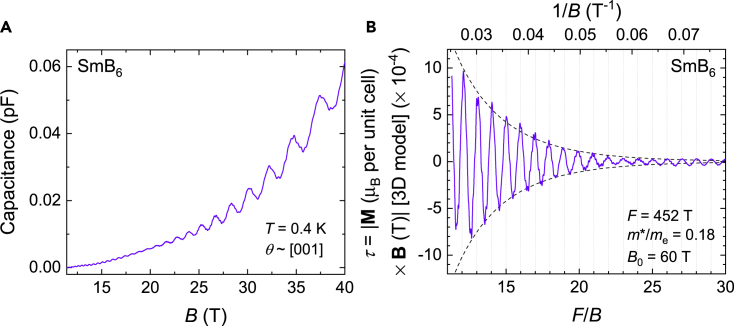
Absolute Amplitude of Magnetic Torque Quantum Oscillations in Pristine Floating Zone-Grown SmB_6_ Single Crystals (A) Quantum oscillations in the magnetic torque of a floating zone-grown single crystal of SmB_6_ are sizable compared to the paramagnetic background, before any background subtraction. Oscillations corresponding to the high frequencies originating from the α branches are also visible. (B) De Haas–van Alphen oscillations in absolute units of magnetic moment corresponding to the *F* = 452 T oscillations after a smooth monotonic background was subtracted. The dashed lines represent the magnetic field dependence of the quantum oscillation amplitude from the impurity scattering (Dingle) damping term for a damping factor *B*_0_ = 60 T.

The size of the low temperature inverse residual resistivity ratio (iRRR) is another indicator of single crystal purity of SmB_6_ (Orendáč et al., 2017; Gabáni et al., 2016; Fuhrman and Nikolić, 2020). Figure 1A compares the resistivity as a function of temperature of the floating zone-grown single crystal SmB_6_ used for our quantum oscillation measurements, the isotopic floating zone-grown crystal of Fuhrman et al. (2018), and crystals grown by various other techniques (Xu et al., 2016; Pristáš et al., 2014; Nickerson et al., 1971). We find that of the various crystal growth methods, single crystals grown by the floating-zone method exhibit the largest values of iRRR. We perform quantum oscillation measurements on floating-zone grown single crystals with the highest values of iRRR identified by extensive screening of over a hundred and thirty samples. Selected floating zone-grown single crystals measured in our quantum oscillation study exhibit an iRRR of order 10^5^, exceeding the iRRR in most other single crystals by over an order of magnitude, and with a value of iRRR/thickness ≈ 400, reflecting the highly insulating bulk contribution. Results of quantum oscillations on these floating-zone grown SmB_6_ were first reported in Tan et al. (2015); more measurements of quantum oscillations on floating-zone grown SmB_6_ are shown in this study (Figures 3 and 4).

### In-Gap Low Energy Excitations in Floating Zone-Grown Single Crystals of SmB_6_

**Figure 5:**
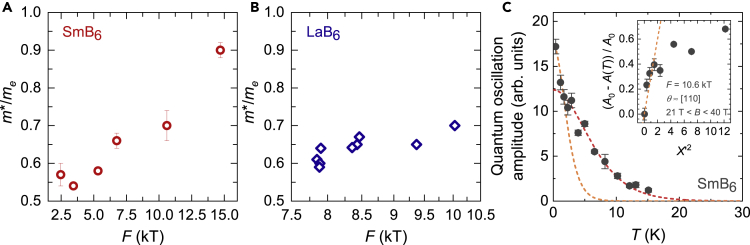
Effective Mass and Quantum Oscillation Temperature Dependence (A and B) Comparison of the quasiparticle effective masses as a function of oscillation frequency (A) for floating zone-grown SmB_6_ measured along [110] (for temperatures down to 1 K) from Hartstein et al. (2018), and (B) for LaB_6_ measured along different field directions based on Ōnuki et al. (1989); Ishizawa et al. (1977); Onuki et al. (1989); Ishizawa et al. (1980); Arko et al. (1976); Suzuki et al. (1987). Error bars in (A) reflect the error from fitting the temperature dependence of quantum oscillation amplitude to the Lifshitz-Kosevich formula. The effective mass of frequencies corresponding to the large α ellipsoids in both materials scale roughly linearly with the oscillation frequency, as expected for Fermi surface sections that originate from the same band. (C) The temperature dependence of quantum oscillation amplitude of the 10.6 kT frequency observed in floating zone-grown SmB_6_ in a magnetic field window of 21–40 T and a temperature range 30 K–550 mK, showing a prominent low-temperature increase. Error bars reflect the FFT noise floor. The inset shows the growth of the 10.6 kT frequency amplitude *A*(*T*) with respect to *A*_0_, the amplitude at the lowest measured temperature, as a function of $X^{\prime 2}$, where $X^{\prime }=2\pi^{2}k_{\text{B}}Tm_{\text{e}}/e\hbar B$, the temperature damping coefficient in the Lifshitz–Kosevich formula, as expected for the Fermi Dirac statistical distribution (Shoenberg, 1984). The low temperature growth in amplitude is linear in $X^{\prime 2}$ (shown by orange dashed line), in contrast to the predicted suppression of temperature-dependent amplitude growth at low temperatures for gapped models. As reported previously in Tan et al. (2015); Hartstein et al. (2018), a deviation from the Lifshitz–Kosevich temperature dependence is seen at the lowest temperatures for the dominant low frequency oscillation, reminiscent of the low temperature upturn of the linear term of specific heat capacity. A similar deviation from the Lifshitz–Kosevich form can also potentially be seen in the high oscillation frequencies; an example using the 10.6 kT frequency is shown here. The red dashed line shows a Lifshitz–Kosevich simulation with effective mass *m*^∗^/*m*_e_ = 0.7 that fits the amplitude as a function of temperature for $T\underset{\approx }{>}1$ K, while the orange dashed line shows a Lifshitz–Kosevich simulation with effective mass *m*^∗^/*m*_e_ = 1.8 that fits the amplitude as a function of temperature for $T\underset{\approx }{<}1$ K.

Unconventional low energy excitations within the insulating gap are established by various experimental findings in floating zone-grown single crystals of SmB_6_ (Tan et al., 2015; Hartstein et al., 2018). Measurements of magnetic torque on floating zone-grown single crystals of SmB_6_ reveal large amplitude quantum oscillations with a multitude of frequencies including high frequencies up to ≈ 15 kT, as shown in Figure 3, corresponding to approximately half the volume of the cubic Brillouin zone. Figure 5C shows the prominent rise in quantum oscillation amplitude of the 10.6 kT frequency at low temperatures, characteristic of low energy excitations that exhibit a Fermi Dirac-like distribution, further brought out in the inset. The low temperature rise in quantum oscillation amplitude contradicts expectations from a gapped model, where the quantum oscillation amplitude would be expected to remain largely flat or to decrease at low temperatures (Knolle and Cooper, 2015). Over a broad temperature range, a deviation from the Lifshitz–Kosevich temperature dependence has previously been reported (Tan et al., 2015; Hartstein et al., 2018). Such a deviation is also potentially seen in high frequency oscillations as suggested by dashed lines in the main panel of Figure 5C.

Furthermore, the linear coefficient of the specific heat capacity has a finite value at low temperatures, providing corroborating evidence for low energy excitations in the gap (Hartstein et al., 2018). Figure 1C shows the measured heat capacity divided by temperature for our floating zone-grown single crystals of SmB_6_, together with comparative measurements of single crystals presented in Fuhrman et al. (2018). Complementary experiments have shown that an excess value of the linear coefficient of specific heat can arise from magnetic impurities (Fuhrman et al., 2018; Gabáni et al., 2001). We find that a finite value of the heat capacity divided by temperature (γ) is seen for pristine floating zone-grown single crystals used in our quantum oscillation measurements, in which ICP-OES and magnetic characterization limit any magnetic impurity concentration to within an upper bound of ≈ 0.04%at. Measured finite values of *γ* ≈ 4 mJ·mol^−1^·K^−2^ (from Hartstein et al. (2018)), and *γ* ≈ 3mJ·mol^−1^·K^−2^ (this study), in the vicinity of 2 K are similar to values measured in isotopically pure floating zone-grown single crystals of SmB_6_ (Fuhrman et al., 2018; Gabáni et al., 2002b), while at least an order of magnitude smaller than the samples impregnated with magnetic impurities (Fuhrman et al., 2018) and off-stoichiometric samples (Orendáč et al., 2017). The measured γ value is also similar to that measured in the metallic LaB_6_ (Stankiewicz et al., 2011). The finite low temperature value of the linear coefficient of specific heat, and the low temperature upturn in the linear coefficient of specific heat resembling the low temperature upturn in quantum oscillation amplitude (Hartstein et al., 2018), are hallmarks of low energy excitations in the highest quality floating-zone grown single crystals of insulating SmB_6_. These features in the linear coefficient of specific heat were also reported in early measurements of isotopically pure floating-zone grown single crystals of SmB_6_ as identifiers of ‘intrinsic coherent state formation within the states at the Fermi energy toward very low temperatures’ (Gabáni et al., 2002b; Flachbart et al., 2006).

### Intrinsic Origin of Quantum Oscillations from the Bulk of Floating Zone-Grown Single Crystals of SmB_6_

In this study, as in our previous study (Tan et al., 2015), quantum oscillation measurements are carried out on floating zone-grown single crystals of SmB_6_ in the 45 T Hybrid magnet at the National High Magnetic Field Laboratory in Tallahassee using a capacitive torque technique (Tan et al., 2015). Complementary techniques have also been used to observe quantum oscillations in these single crystals, including Faraday magnetometry in a superconducting magnet at the Institute for Solid State Physics, University of Tokyo, and magnetic susceptibility in a 65 T pulsed magnet at the National High Magnetic Field Laboratory in Los Alamos (Hartstein et al., 2018). Figures 3A–3C shows measured oscillations in the magnetic torque of floating zone-grown SmB_6_ from the same batch of crystals as the ones employed for the tests of sample quality detailed earlier, and the corresponding oscillation frequencies as found by Fourier transform. Similar to our previous measurements (Tan et al., 2015), the strongest frequency at low magnetic fields is 500 T, with high frequencies appearing with increasing field; the highest frequency is 15 kT, which appears above applied fields of 30 T. We find large amplitude quantum oscillations comparable to the paramagnetic torque background (Figure 4). The large size of quantum oscillations indicates an origin that is not from just a minute fraction of the sample but rather corresponds to the bulk of the sample.

We make a quantitative comparison between the absolute size of the measured quantum oscillations and the theoretical amplitude expected for bulk de Haas–van Alphen oscillations within the Lifshitz–Kosevich theory. We convert the measured capacitive torque to absolute units of magnetic moment by using the spring constant of the cantilever, as detailed in Hartstein et al. (2018). We have cantilever length *L* = 3.8 mm, distance between cantilever and fixed Cu plate *d*_0_ = 0.1 mm, spring constant *k* = 30 N·m^−1^, unit cell volume $V_{\text{u}.\text{c}.}=a_{\text{u}.\text{c}.}^{3}=0.07{\text{ nm}}^{3}$, and crystal volume *s*^3^ = 0.49 mm^3^. We thus convert the measured torque magnetization in terms of capacitance (*C*) to an absolute magnetic moment *p*_*s*_ in units of Bohr magneton per unit cell by the expression:

(Equation 1) $$\text{Δ}p_{s}=\frac{0.175}{B}\cdot \text{Δ}C\;\text{T}\cdot {\text{pF}}^{-1}\mu_{\text{B}}\text{ per unit cell.}$$

The measured quantum oscillatory magnetic moment converted to absolute units for a typical magnetic field sweep is shown in Figure 4B. We estimate the theoretical amplitude of the intrinsic quantum oscillatory magnetic moment perpendicular to the applied magnetic field in units of Bohr magneton per unit cell for a three-dimensional Fermi surface using the Lifshitz–Kosevich formula:

(Equation 2) $$p_{s}=D\cdot R_{T}R_{D}R_{S}\cdot \text{sin}\left(2\pi F/B+\varphi \right)\cdot \text{sin}\theta_{M}\text{,}$$

where sin(2πF/B+φ) is the oscillatory term, *θ*_*M*_ is the angle between the magnetic field *B* and the total magnetic moment, and *R*_*T*_, *R*_*D*_, and *R*_*S*_ are damping terms due to finite temperature, impurity scattering, and spin-splitting. The exponential damping term *R*_*D*_ is expressed as $R_{D}=\text{exp}\left(-B_{0}/B\right)$, with damping factor *B*_0_ for each frequency. *D* is the infinite field, zero spin-splitting amplitude given by:

(Equation 3) $$D=f\left(r\right)\frac{m_{\text{e}}}{m^{\text{∗}}}{\left(\frac{a_{\text{u}.\text{c}.}k_{\text{F}}}{\pi }\right)}^{3}\sqrt{\frac{B}{8F}}\text{,}$$

where *f*(*r*) is the anisotropy term, *m*^∗^ is the effective mass, *k*_F_ is the Fermi wavevector, and *F* is the oscillation frequency. For the *F* = 452 T frequency oscillations shown in Figure 4B, we have *m*^∗^*/m*_e_ = 0.18, *B*_0_ = 60 T, a degeneracy of two and *f*(*r*) = 0.5 based on the ellipsoidal model we fit with in Figure 7A, and estimate *R*_*S*_ = 0.5–1, sin*θ*_*M*_ = 0.1–1. The resulting estimate for the theoretical amplitude of quantum oscillations from a three-dimensional Fermi surface is $\approx \;10^{-3}–10^{-2}\;\mu_{\text{B}}\cdot$ T per unit cell at *B* = 30 T, whilst the measured quantum oscillation amplitude considering a bulk origin is $\approx 10^{-3}\;\mu_{\text{B}}\cdot$ T per unit cell, showing consistency with the theoretical estimate for bulk oscillations and the measured size of the oscillations within an order of magnitude. Crucially, the measured quantum oscillation amplitude assuming an origin from only the crystal surface would be at least three orders of magnitude larger than the theoretically expected quantum oscillation amplitude for a two-dimensional Fermi surface (see Hartstein et al. (2018) for a calculation), making this scenario implausible. The same comparison for the high frequencies is hindered by the imprecise estimate of a much higher damping factor due to only high Landau levels being accessed, and requires higher magnetic fields for a more accurate comparison to be made.

### Comparison of Quantum Oscillations in Floating Zone-Grown Single Crystals of SmB_6_ with Quantum Oscillations in Metallic LaB_6_

**Figure 6:**
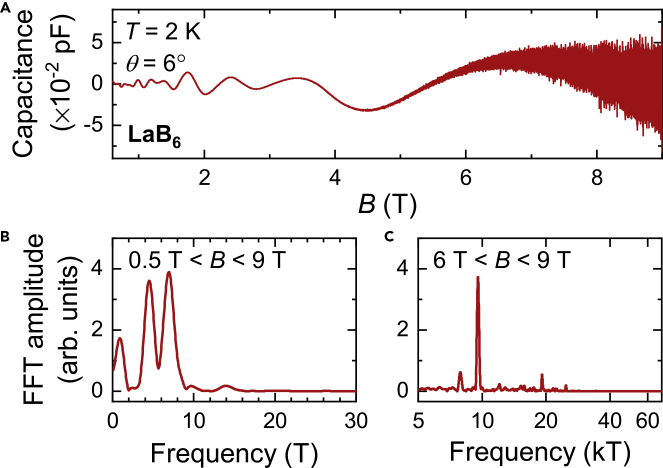
Magnetic Torque Quantum Oscillations in Pristine Floating Zone-Grown LaB_6_ Single Crystals (A) Quantum oscillations in the magnetic torque of floating zone-grown single crystals of LaB_6_ measured in this study as a function of magnetic field, exhibiting both low- and high-frequency oscillations. The field is aligned 6° from the [001] direction in the [001]-[111]-[110] rotational plane. (B and C) Fourier transforms of the measured magnetic torque as a function of inverse magnetic field in the field ranges (B) 0.5 T < *B* < 9 T and (C) 6 T < *B* < 9 T, revealing multiple quantum oscillation frequencies up to 10 T corresponding to the ρ branches, and between 8 and 10 kT corresponding to the α branches (see Figure 7B).

In this study, we perform new quantum oscillation measurements on pristine floating-zone grown single crystals of metallic LaB_6_ to compare with quantum oscillations in SmB_6_ (Figures 6A–6C). We find that quantum oscillations in insulating SmB_6_ show a surprisingly similar overall behavior to quantum oscillations in metallic LaB_6_. A broad range of oscillation frequencies is seen in LaB_6_, which are observed down to lower fields due to a lower Dingle temperature as compared to SmB_6_. The size of the observed large quantum oscillations in metallic LaB_6_ is comparable to expectations for bulk oscillations from the Lifshitz–Kosevich theory (Hartstein et al., 2018), as expected for a metal. The surprising similarity of quantum oscillations in bulk insulating SmB_6_ to its nonmagnetic metallic counterpart LaB_6_ suggests that the bulk Fermi surface in bulk insulating SmB_6_ mimics the conduction band Fermi surface. Any theoretical explanation that seeks to explain the intrinsic bulk quantum oscillations observed in bulk insulating SmB_6_ must explain this intriguing Fermi surface similarity.

**Figure 7:**
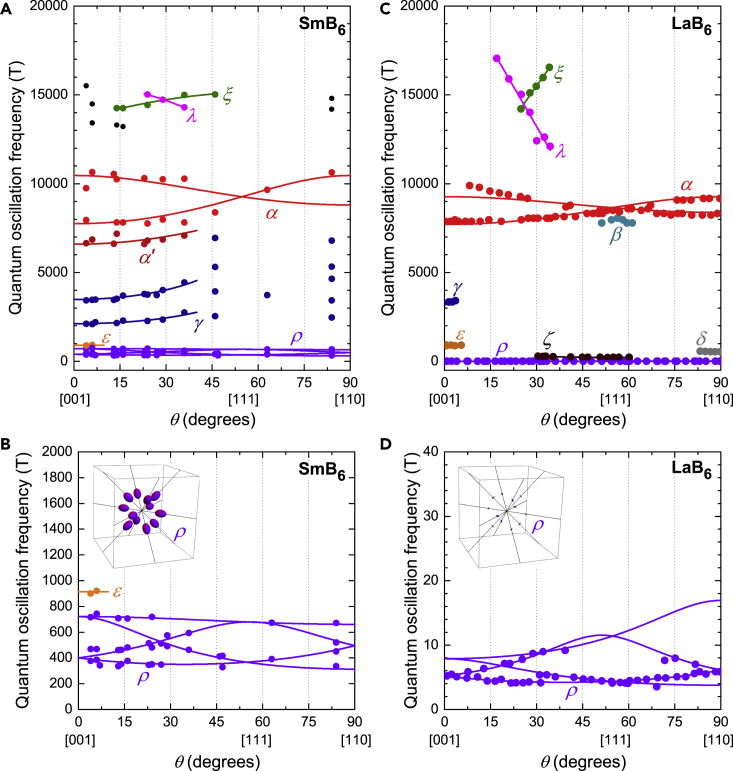
Comparison of Angular Dependence of Quantum Oscillation Frequencies in SmB_6_ and LaB_6_ Angular dependence of the quantum oscillation frequencies in the [001]-[111]-[110] rotation plane measured for floating zone-grown single crystals of (A and B) SmB_6_ from Tan et al. (2015), and (C and D) LaB_6_ from Ōnuki et al. (1989); Ishizawa et al. (1977), both in good agreement with the angular dependence of a three-dimensional ellipsoidal Fermi surface characteristic of metallic hexaborides. (A) and (C) show the similarity of the higher frequency branches, particularly the main α branches, which correspond to ≈50% of the Brillouin zone volume when the three-fold degeneracy is included, while (B) and (D) compare the lower frequency ρ branches, which give rise to the most prominent oscillations, together with illustration of the corresponding Fermi surface sections. Solid lines are based on a three-dimensional ellipsoidal Fermi surface model. For the α branches of SmB_6_, we obtain a minimum frequency of *F*_min_ = 7580 T and a ratio of the semi-principal axes of 1.4. For the α branches of LaB_6_ we obtain *F*_min_ = 7710 T, and a ratio of the semi-principal axes of 1.2. Fitting to the ρ branches of SmB_6_ we find a minimum frequency of *F*_min_ = 313 T, and the ratios of the two longer semi-principal axes to the shortest axis to be 2.3 and 1.1. In the case of LaB_6_, we find *F*_min_ = 3.7 T and ratios of 3.5 and 1.5.

The angular dependence of the quantum oscillation frequencies for floating zone-grown SmB_6_ is shown in Figures 7A and 7B. Measured quantum oscillation frequencies and angular dependence in floating zone-grown SmB_6_ show similarities with the metallic rare-earth hexaborides (Ōnuki et al., 1989), which crystallize in a cubic crystal structure similar to SmB_6_. Figure 7 shows a comparison of the angular dependence of the quantum oscillation frequencies in insulating SmB_6_ with quantum oscillation frequencies in metallic LaB_6_. We see a commonality over the entire frequency range with corresponding frequency branches of all major Fermi surface sections. Importantly, we see the high-frequency α branch across the entire angular range for both SmB_6_ and LaB_6_, corresponding to the dominant section of the Fermi surface. We also observe the high-frequency λ and ξ branches for both materials, which appear for a specific angular range, where two α orbits join through the small neck area (Ishizawa et al., 1977). These high-frequency branches are observed across the metallic hexaborides (Ōnuki et al., 1989), and, as demonstrated below, are not found in aluminum. The high-frequency branches constitute more than 90% of the Fermi surface sections of the ellipsoidal Fermi surface of metallic hexaborides. As first presented in Tan et al. (2015), Figures 7A and 7B shows the angular dependence of quantum oscillations measured in SmB_6_ compared with the three-dimensional ellipsoidal Fermi surface used to model the Fermi surface in the metallic hexaborides (Ōnuki et al., 1989; Ishizawa et al., 1977; Harrison et al., 1993). The size of the small connecting ellipsoids, and therefore the ρ frequencies, varies with subtleties of the Fermi surface geometry, and is found to vary between the hexaboride materials.

Measured quantum oscillation frequencies in SmB_6_ are characterized by similarly light measured quasiparticle effective masses compared to the conduction electron Fermi surface of LaB_6_ (Figure 5). For frequencies originating from the large α ellipsoids, corresponding to the main cross sections (8 kT $\underset{\approx }{<}F\underset{\approx }{<}11$ kT), and hole-like orbits that appear for certain angular ranges ($F\underset{\approx }{>}800$ T), the effective masses of SmB_6_ scale linearly with the oscillation frequency, very similar to that observed for LaB_6_. Such a relation between the effective mass and the oscillation frequency is indicative of Fermi surface sections that originate from the same band, a further addition to a suite of evidence for a three-dimensional ellipsoidal Fermi surface from the intrinsic bulk of the material.

### Distinguishing Intrinsic Bulk Quantum Oscillations in Pristine SmB_6_ from Those due to Aluminum Flux Inclusions

**Figure 8:**
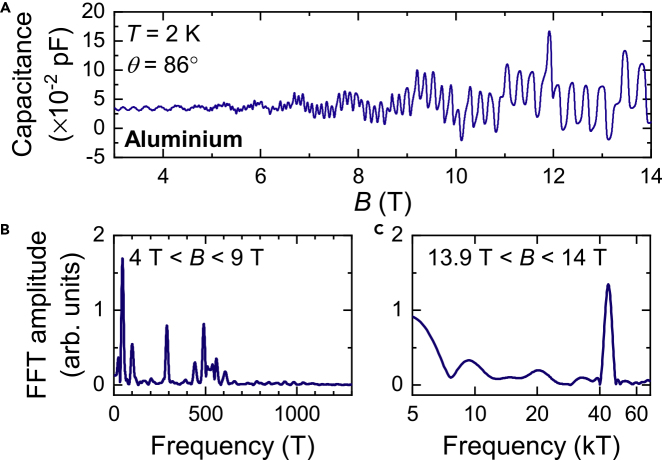
Magnetic Torque Quantum Oscillations in Single Crystals of Elemental Aluminum (A) Quantum oscillations in the magnetic torque of Aluminum single crystals measured in this study as a function of magnetic field. The field is aligned 86° from the [001] direction in the [001]-[111]-[110] rotational plane. (B and C) Fourier transforms of the measured magnetic torque as a function of inverse magnetic field in the field ranges (B) 4 T < *B* < 9 T and (C) 13.9 T < *B* < 14 T, revealing multiple frequency peaks up to 500 T, and a very high frequency peak of 40 kT, when taking a narrow field window, corresponding to the ψ branch (see Figure 9B).

Recently, a comparison of quantum oscillations in single crystals of aluminum was made with quantum oscillations in Aluminum flux-grown single crystals of SmB_6_ (Thomas et al., 2019). Given that the floating zone-grown single crystals of SmB_6_ used for our measurements are shown to be free of aluminum down to the detection limit of ≈ 0.01%at by bulk ICP-OES measurements, quantum oscillations in these single crystals are established to be intrinsic to bulk SmB_6_. These floating zone-grown single crystals of SmB_6_ thus provide an excellent model system to examine ways in which intrinsic quantum oscillations in SmB_6_ can be distinguished from quantum oscillations originating from metallic aluminum. In order to make this comparison, we present new quantum oscillation measurements in pristine single crystals of aluminum in this study (Figures 8A–8C).

**Figure 9:**
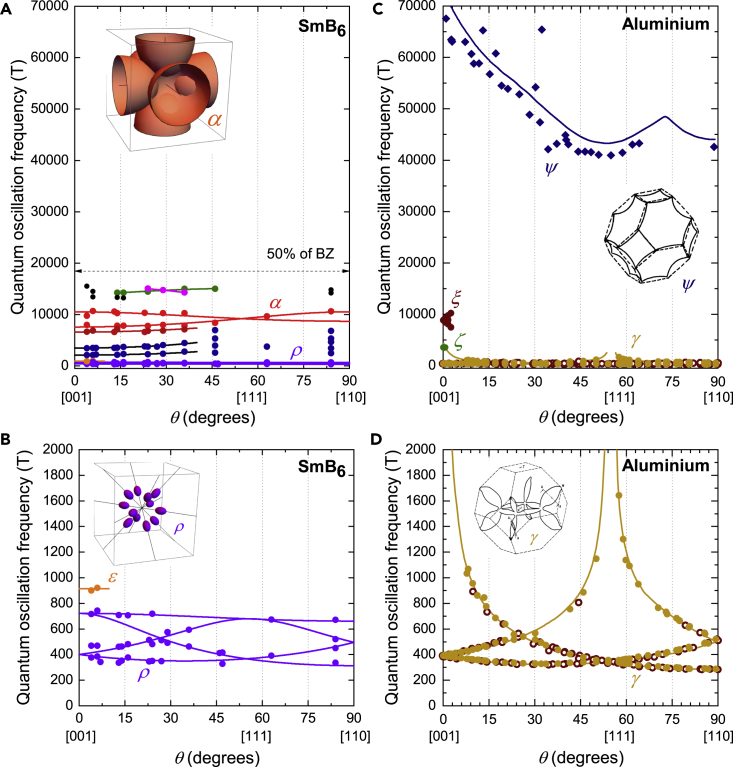
Comparison of Angular Dependence of Quantum Oscillation Frequencies in SmB_6_ and Elemental Aluminum Comparison on the same frequency scale of the measured angular dependence of quantum oscillation frequencies in the [001]-[111]-[110] rotation plane of floating zone-grown SmB_6_ (A and B) from Tan et al. (2015), with aluminum (C and D) from this study, Anderson and Lane (1970); Larson and Gordon (1967), and Boyne and Mackinnon (1978). The comparison of the high frequency branches in (A) and (C) shows a multitude of branches between 2 kT and 16 kT spanning the entire angular range for SmB_6_, but none for aluminum, which has a prominent high-frequency branch above 40 kT (circles are measured by this study, diamonds are from Anderson and Lane (1970), and squares are from Larson and Gordon (1967); Boyne and Mackinnon (1978)). The arrow denotes the maximum expected frequency for half-filling assuming a single spherical Fermi surface section, above which we would not expect to see frequencies for a metallic equivalent, similar to LaB_6_. (B) ρ branches found for SmB_6_ have a flat angular dependence corresponding to a Fermi surface of twelve ellipsoids along <110>, as shown in the illustration. (D) Frequencies for aluminum measured in this study show good overlap with previous measurements (brown circles (Larson and Gordon, 1967; Boyne and Mackinnon, 1978)), and trace the γ branches to much higher frequencies than previous measurements, with frequencies as high as 1600 T. A pronounced divergence of the measured frequencies is seen in the [110]-[001] rotation plane, corresponding to a necklace-like Fermi surface of elongated arms as shown in the illustration (taken from Harrison (1959)). Solid lines follow Ashcroft's model (Ashcroft, 1963) and are from Larson and Gordon (1967). Frequency points in Boyne and Mackinnon (1978) corresponding to harmonics are not shown. Illustrations of Fermi surface sections corresponding to the α and ρ sections in SmB_6_ are from Tan et al. (2015), while those corresponding to the ψ and γ sections in aluminum are from Harrison (1959).

Figure 9 shows the angular dependence of the quantum oscillation frequencies observed for aluminum. The high and intermediate quantum oscillation frequencies above 2 kT observed in floating zone-grown single crystals of SmB_6_ are dramatically different from the high quantum oscillation frequencies observed in single crystals of aluminum. While the low frequency quantum oscillations (ρ frequencies in SmB_6_ and the γ frequencies in aluminum) have some similar features in the range between 300 T and 500 T, differences become apparent on considering the full angular dependence. The elongated necklace-like Fermi surface of aluminum yields divergent frequencies along [001] and [111], unlike the three-dimensional ellipsoidal Fermi surface identified in our floating zone-grown single crystals of SmB_6_, which yields a flat continuum of quantum oscillation frequencies at all angles. In contrast, divergent frequencies along several symmetry directions were reported in aluminum flux-grown single crystals of SmB_6_ in Li et al. (2014). A major difference between quantum oscillations observed for floating zone-grown samples of SmB_6_ and for aluminum is the appearance of intermediate frequency branches (2–15 kT) in SmB_6_, corresponding to the main Fermi surface sections, that have no parallels in aluminum. The intermediate frequencies in SmB_6_ contrast a non-degenerate 40–80 kT branch in elemental aluminum, a detailed characterization of which was not available previously, that in turn has no parallels in SmB_6_. Large amplitude oscillations, the intermediate frequency branches (2–15 kT) and the identification of the four flat branches of the ρ frequencies are clear signatures of intrinsic bulk quantum oscillations in SmB_6_ corresponding to a three-dimensional Fermi surface.

The similarity at the lower end of the frequency branches starting at approximately 300 T between bulk quantum oscillations intrinsic to pristine floating zone-grown SmB_6_ and quantum oscillations in metallic aluminum is surprising at first sight. One might consider whether the similarly sized Fermi surfaces are a consequence of the fact that aluminum and SmB_6_ both have cubic lattice symmetries, nearly matching lattice constants (*a* = 4.13 Å in the case of SmB_6_, and *a* = 4.05 Å in the case of aluminum) and the same number of valence electrons. Under these conditions some similarity in the quantum oscillation frequencies might be expected from Luttinger's theorem alone. However, most importantly, the comparison between quantum oscillations intrinsic to floating zone-grown SmB_6_ and quantum oscillations from metallic aluminum enables us to clearly outline major differences between these superficially similar quantum oscillations. Such a comparison was not previously possible in Thomas et al. (2019) or Li et al. (2014) which relied on measurements in aluminum flux-grown single crystals of SmB_6_.

## Discussion

We have established intrinsic bulk quantum oscillations in pristine floating zone-grown single crystals of the bulk Kondo insulator SmB_6_, thus establishing a new class of unconventional insulators, following the first report in Tan et al. (2015). Our findings bring to the fore the question of how such bulk quantum oscillations can arise in a bulk insulator given that thus far they have been considered the preserve of metals. This striking phenomenon first observed in SmB_6_ (Tan et al., 2015), now extends across a growing class of unconventional insulators including YbB_12_ (Xiang et al., 2018; Liu et al., 2018), and overturns the previously held belief that quantum oscillations are a property unique to metals. A theoretical challenge is thus posed that requires an understanding beyond our current interpretation of quantum oscillations that occur only in metals. The study of more materials in the class of unconventional insulators will provide further clues as to the new theoretical paradigm underlying this rich new phenomenon.

### Limitations of the Study

SmB6 is the first of a new class of unconventional bulk insulators that display quantum oscillations. This phenomenon will be better understood when more materials in the class of unconventional bulk insulators are identified.

### Resource Availability

#### Lead Contact

Further information and requests for resources and reagents should be directed to and will be fulfilled by the lead contact, Suchitra E. Sebastian (suchitra@phy.cam.ac.uk).

#### Materials Availability

This study did not generate new unique reagents.

#### Data and Code Availability

The data that support the plots within this paper and other findings of this study are available from the lead contact upon reasonable request.

## Methods

All methods can be found in the accompanying Transparent Methods supplemental file.

## Supplemental Information

Document S1. Transparent Methods
