# Supplementary material for: Intrinsic Bulk Quantum Oscillations in a Bulk Unconventional Insulator SmB6
Source: iScience. 2020 Oct 1;23(11):101632. doi: 10.1016/j.isci.2020.101632 (PMC7593550; doi:10.1016/j.isci.2020.101632)
Supplement: Document S1. Transparent Methods [file mmc1.pdf]

iScience, Volume *23*

Supplemental Information

Intrinsic Bulk Quantum Oscillations in a Bulk Unconventional Insulator SmB_6

Máté Hartstein, Hsu Liu, Yu-Te Hsu, Beng S. Tan, Monica Ciomaga Hatnean, Geetha Balakrishnan, and Suchitra E. Sebastian

Supplemental information for

Intrinsic bulk quantum oscillations in a bulk unconventional insulator SmB_6

Máté Hartstein, Hsu Liu, Yu-Te Hsu, Beng S. Tan, Monica Ciomaga Hatnean, Geetha Balakrishnan, & Suchitra E. Sebastian

Correspondence to: suchitra@phy.cam.ac.uk

This PDF file includes:

Transparent methods

Supplemental reference list

Transparent methods

Sample preparation

Single crystals of SmB_6 and LaB_6 were grown using the floating zone method in an image furnace using 99.9% purity SmB_6 powder from Alfar Aesar and 99.9% LaB_6 powder from Cerac (Hatnean et al. 2013, Balakrishnan et al. 2003). Single crystals were oriented using a Multiwre X-ray Laue backscattering camera, then cut using a 25 µm tungsten wire saw into rectangular cuboids followed by electropolishing with dilute acid. The dominant faces of the rectangular cuboids were aligned perpendicular to either the [001] or [110] high symmetry axes. Elemental Aluminum was grown from the melt using radio-frequency melting techniques.

Mass spectroscopy

Chemical impurity analysis was performed with inductively coupled plasma optical emission spectrometry (ICP-OES) by Exeter Analytical UK Ltd. Samples from the same growth batches as those used in quantum oscillation measurements were used, with a total mass of 50 mg to allow for a broad multi-element scan with high levels of precision. The samples were digested in a nitric acid matrix using microwaves, and introduced to the ICP-OES along with internal standards to aid precision. The large quantity of sample used $\approx$ 50 mg enables higher detection accuracy and precision across a broad range of elements examined than had been previously reported. Extrinsic impurity content other than Tellurium and Yttrium is ruled out to within the detection limit ($\approx$ 0.01%at in most cases). The full results are shown in Table 1.

Magnetization measurements

Magnetization measurements shown in Fig. 1(b) were taken as a function of both temperature and applied magnetic field at the Institute of Solid State Physics of the University of Tokyo. The sample magnetization as a function of applied magnetic field is fitted with a Langevin function

to approximate the level of magnetic impurity content. The magnetization per mole is fit by:

$$M(H,T) = \chi_{\text{bulk}} H + c_{\text{impurity}} \mu_{\text{eff}} \left(\coth \left(\frac{\mu_{\text{eff}} H}{k_{\text{B}} T} \right) - \frac{k_{\text{B}} T}{\mu_{\text{eff}} H} \right), \tag{1}$$

where χ_{bulk} is the intrinsic bulk susceptibility, H is the applied magnetic field, c_{impurity} is the impurity concentration, k_{B} is the Boltzmann constant, and T is temperature. The effective magnetic moment, μ_{eff}, was taken as 7.94 μ_{B}, corresponding to the Gd^{3+} state, following Fuhrman et al. (2018). The fit limits any magnetic impurity content to $\approx 0.04\%$at.

Torque magnetometry

Torque magnetometry measurements were performed in DC magnetic field facilities at the National High Magnetic Field Laboratory, Tallahassee, Florida, USA using the 45 T hybrid magnet and a 35 T resistive magnet. The hybrid magnet was fitted with a ^{3}He insert, while the 35 T resistive magnet was fitted with a dilution fridge insert. Torque measurements of LaB_6 and elemental Aluminum were performed in Quantum Design Inc. Physical Property Measurement Systems capable of generating applied magnetic fields of 9 T or 14 T.

Cantilevers were laser-cut from a 20 μm thin non-magnetic BeCu into flexible T-shaped pieces, with a narrow tail anchored on an electrical lead and a wide rectangular head floating on top of a fixed Cu baseplate. The cantilever and the Cu baseplate form the two plates of a capacitor, whose capacitance change was measured by a General Radio analogue capacitance bridge in conjunction with a phase sensitive detector.

This configuration of cantilever and base plate was mounted on a rotatable housing unit capable of rotating through 360° in a plane parallel to the applied magnetic field. A Hall probe was used to determine the angle of rotation. Proximity to highly symmetric crystallographic directions was verified by vanishing torque signals with a change of sign of the magnetic torque background on either sides.

References

Balakrishnan, G., Lees, M. & Paul, D. M. (2003), ‘Growth of large single crystals of rare earth hexaborides’, *Journal of crystal growth* **256**(1-2), 206–209.

Fuhrman, W., Chamorro, J., Alekseev, P., Mignot, J.-M., Keller, T., Rodriguez-Rivera, J., Qiu, Y., Nikolić, P., McQueen, T. & Broholm, C. L. (2018), ‘Screened moments and extrinsic in-gap states in samarium hexaboride’, *Nature Communications* **9**(1), 1539.

Hatnean, M. C., Lees, M. R., Paul, D. M. & Balakrishnan, G. (2013), ‘Large, high quality single-crystals of the new Topological Kondo Insulator, SmB_6’, *Scientific Reports* **3**, 3071.
